# P-18. Incidence of Pneumococcal Disease and Vaccination Coverage among US Adults by Age and Risk Groups

**DOI:** 10.1093/ofid/ofae631.226

**Published:** 2025-01-29

**Authors:** M Doyinsola Bailey, Yi-Ling Huang, Lei Ai, Salini Mohanty, Valina C McGuinn, Kelly D Johnson, Nicole Cossrow

**Affiliations:** Merck & Co., Inc., Rahway, NJ, USA, Philadelphia, Pennsylvania; Merck, Boston, Massachusetts; Merck, Boston, Massachusetts; Merck & Co., Inc, Rahway, New Jersey; Merck, Boston, Massachusetts; Merck & Co., Inc, Rahway, New Jersey; Merck & Co, Inc., Kenilworth, New Jersey

## Abstract

**Background:**

Older adults and those with underlying medical conditions are at increased risk for illness, hospitalization, and death from pneumococcal disease (PD). The CDC currently recommends pneumococcal vaccination for adults ≥65 years and adults ≥19 years with underlying medical conditions who are at greater risk of PD. Identifying differences in pneumococcal vaccination coverage (VCR) and disease incidence is necessary to develop and implement strategies to improve VCR. We estimated VCR and incidence of PD, by age and risk group, among adults ≥18 years in the United States from 2017-2020.

**Figure:**
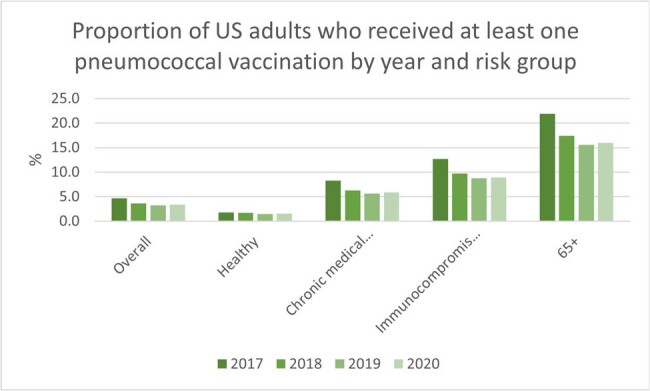

**Methods:**

This was a retrospective study of administrative claims data from Merative® MarketScan® Commercial Claims and Encounters Database. From 2017-2020, annual incidence rates of invasive pneumococcal disease (IPD), non-bacteremic pneumococcal pneumonia (NBPP) and all-cause pneumonia (ACP) were estimated by age group (18-49, 50-64, ≥65 years) and risk group (healthy, with chronic medical conditions (CMC) and with immunocompromising conditions (IC)) among adults with at least 2.5 years of continuous enrollment. Adults were considered vaccinated if they had at least one pneumococcal vaccine within 2 prior years to their index date. The percentage of pneumococcal vaccination was reported annually from 2017-2020.

**Figure:**
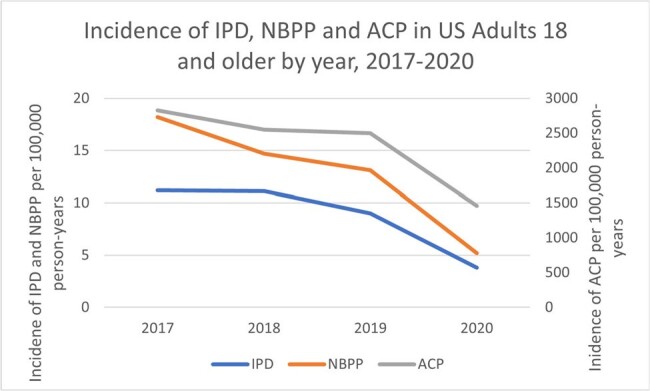

**Results:**

From 2017-2020, there were approximately 9-10 million patients annually in the study population. Less than 25% of US adults recommended for pneumococcal vaccination were vaccinated (Figure 1). Vaccination rates were highest among those ≥65 years (Figure 1). Annual incidence rates per 100,000 person-years decreased from 2017 to 2020 for IPD, NBPP and ACP (Figure 2). Each year, the incidence of PD was consistently higher among those ≥65 years, and those with IC or CMC. There was a decrease in the incidence of PD in 2020, coinciding with the COVID-19 pandemic.

**Conclusion:**

Despite ACIP recommendations, vaccination rates remain low in those recommended to receive a pneumococcal vaccine, specifically among older adults and those with underlying conditions. This analysis underscores the opportunity to improve VCR and prevent PD in US adults, especially those at greatest risk.

**Disclosures:**

**M. Doyinsola Bailey, PHD, MPH**, Merck & Co., Inc., Rahway, NJ, USA: Employee|Merck & Co., Inc., Rahway, NJ, USA: Stocks/Bonds (Public Company) **Yi-Ling Huang, PhD**, Merck: Stocks/Bonds (Public Company) **Salini Mohanty, DrPH, MPH**, Merck & Co., Inc.: Employee|Merck & Co., Inc.: Stocks/Bonds (Public Company) **Kelly D. Johnson, PhD**, Merck & Co., Inc.: Employee|Merck & Co., Inc.: Stocks/Bonds (Public Company) **Nicole Cossrow, PhD**, Merck: Stocks/Bonds (Public Company)

